# Hepatitis B and C Viruses and Hepatocellular Carcinoma

**DOI:** 10.3390/v2081504

**Published:** 2010-07-27

**Authors:** Birke Bartosch

**Affiliations:** 1 INSERM, U871, 69003 Lyon, France ; E-Mail: Birke.Bartosch@inserm.fr; Tel.: +33-472-681-975; Fax: +33-472-687-070; 2 Université Lyon 1, IFR62 Lyon-Est, 69008 Lyon, France; 3 Hospices Civils de Lyon, Hôtel Dieu, Service d’hépatologie et de gastroentérologie, 69002 Lyon, France

## Introduction

**Figure 1.**
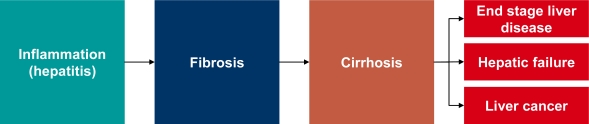
Liver disease progression overview.

Chronic liver disease is responsible for over 1.4 million deaths annually [1] and is characterized by permanent inflammatory processes that predispose to liver cancer and in particular hepatocellular carcinoma (HCC). In healthy liver, inflammatory processes stimulate growth and repair and restore normal liver architecture. However, if liver inflammation becomes chronic, the balance of damage *versus* regeneration in the liver is disrupted and can lead to the formation of excessive scar tissue, termed fibrosis. In the long-term, an exacerbation of fibrosis will lead to cirrhosis, which is characterized by abnormal liver architecture and function and is associated with a significant reduction in overall health and wellbeing. At cirrhotic stages, liver damage is often irreversible or difficult to treat. Cirrhosis leads frequently to death from liver failure or to HCC (Figure 1). Indeed, HCC is the first cause of death in cirrhotic patients [2], and is a tumor with poor prognosis, ranking third in terms of death by cancer. Furthermore, it is the fifth most prevalent cancer worldwide, with 800,000 new cases per year in the world [2,3].

**Figure 2.**
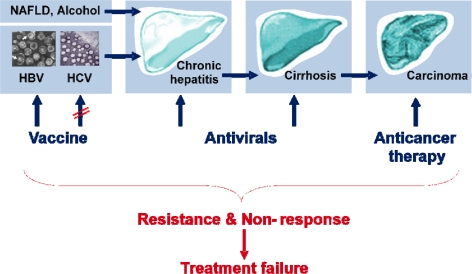
Liver disease progression and treatment options.

An estimated 75% of all HCC cases are due to chronic infection with hepatitis B (HBV) or hepatitis C (HCV) viruses, and the incidence rate of HCC is predicted to increase in western countries until the 2020s due to HCV infection [3,4]. Currently available HCC therapies, based on anticancerous agents, chemotherapy, surgical or radiation-based resection, are inefficient, mainly due to usually late diagnosis and high recurrence rates after surgical resection, and usually end with treatment failure (Figure 2). Liver transplantation also remains a difficult strategy in patients with HCC. Thus, prevention of HCC by treating and preventing HBV and HCV infection – the major causative agents of HCC – is of great medical importance, particularly in the light of 400 million chronic HBV and 170 million chronic HCV carriers worldwide [3]. The fact that a major part of the worldwide HCC cases is due to infection with HBV or HCV warrants a better understanding of the life cycle and host-cell interactions of these two viruses in order to allow the design of new rationalized therapeutic strategies aiming at improving prevention and patients’ treatment. Major issues in the future will be to answer key questions in the respective HCV, HBV and HCC fields in order to gain a comprehensive view of the situation: Why are these viruses so successful at establishing chronic infections and how do they modulate and escape innate and adaptive host-immune responses to do so? How can we restore efficient antiviral immunity and eliminate or control chronic infection? How can we prevent disease progression and treat HCC? This special issue discusses recent advances in our knowledge of the basic biology of HBV and HCV, how these viruses may establish chronic infection and cause liver damage and ultimately cancer in the long-term. Finally, an update of the treatment and therapeutics that are in development against these major human pathogens is given.

### Current limitations to the treatment of HBV and HCV

Although a vaccine exists against HBV, it is not always available in developing countries for economical reasons. Antiviral treatments against HBV only keep the viral load low but do not eliminate the virus and thus favor the emergence of drug-resistant mutants, as discussed in detail in by Lampertico *et al*. and De Clercq *et al*. in reviews that focus on antiviral-treatment and -development, respectively. Concerning HCV, no vaccine will become available in the immediate future and the outlook seems dull, despite important progress over the last few years, as reviewed by M. Major. The therapeutic arsenal against HCV is based upon the use of interferon and ribavirin, a costly and difficult treatment to support, to which a significant proportion of the patients do not respond. With the development of novel antivirals that directly interfere with HCV, treatment options are advancing and treatment success is improving. However, despite the novel combination therapies that are now becoming available, and which are reviewed in detail by Delang *et al.* in this issue, overall viral resistance and non-response to treatment will remain critical issues for treatment of both HBV and HCV patients (Figure 2) [5,6]. Thus, a better understanding of the basic biology of these viruses and the development of novel therapeutic approaches remains an important issue.

### Chronic HBV and HCV infection and the pathological consequences

Hepatitis B and C virus are members of two different viral families, but both display a strong hepatotropism, whose underlying molecular mechanisms are not yet entirely understood. HBV belongs to the family of *hepadnaviridae*, and Schaedler *et al.* give an overview of its life cycle in this issue. HBV contains a partially double stranded genomic DNA that does not encode a dominant oncogene. Thus, HBV is thought to be carcinogenic in an indirect fashion, such as by insertional activation of cellular oncogenes, the induction of genetic instability upon HBV DNA integration or by the regulatory protein HBx as well as by modulation of host immune reponses [7].

HCV is member of the *flaviviridae*, where it forms its own genus, Hepacivirus. HCV is a small, enveloped positive-sense, single stranded RNA virus, and its life cycle is thought to be predominantly cytoplasmic [8]. Thus, HCV is likely to predispose to cancer by alteration of cell signaling and metabolism as well as by modulating and inducing immune responses [9]. Modulation of cellular immunity and metabolism are processes that are likely to establish a liver microenvironment that is characterized by chronic inflammation, oxidative stress and repair processes that lead to liver fibrosis, cirrhosis and HCC over time [10]. In addition, some HCV proteins have been shown to alter cell proliferation and cell cycle checkpoint machineries as well [11,12].

The fact that both HCV and HBV manage to escape elimination by host innate and adaptive immune responses has been a focus of intensive research in many laboratories. Indeed, innate as well as adaptive immune responses of the host have been shown to be compromised by both viruses, albeit by different mechanisms. For a long time, HBV has been thought not to induce innate immunity at all. In this issue, Ait-goughoulte *et al.* discuss recent data sets obtained by several different laboratories that suggest that HBV is not necessarily a stealth virus, but can be sensed by the innate immune system and has rather evolved ways to blunt these responses. In respect to HCV, its ability to evade innate immunity at several levels *in vitro* has been well acknowledged in the field, and a review by Markus Heim in this issue presents the underlying molecular mechanisms.

The precise role of adaptive immune responses in the clinical outcomes of HBV and HCV infection are still only partially defined. Regarding HCV, recent studies suggest that the nature of the adaptive immune responses during the acute phase determine whether the virus is cleared or whether a chronic infection manifests itself. Two reviews, by Zeisel *et al.* and Bengtsch *et al.*, summarize different aspects of the adaptive immune responses as determinants of the outcome of HCV infection and outline current concepts of the evasion strategies HCV may use to prevent its elimination. Unravelling these important mechanisms of virus-host interaction will contribute to the development of novel strategies to prevent and control HCV infection. Similarly to HCV infection, successful control of HBV infection requires activation of several arms of the adaptive immune system including B cells, helper and cytotoxic T cells. In addition, the role of the intrahepatic environment has been shown to modulate virus-specific immunity and has therefore become an important focus of research. Bertoletti *et al.* discuss how the liver environment and the network of immune as well as parenchymal and non-parenchymal cells interact and communicate to achieve HBV clearance.

Chronic activation of the immune system, as it is the case for HCV and HBV infections, creates oxidative stress via cytotoxic degradation of infected or damaged cells. It has been known for some time that HCV replication aggravates the oxidative microenvironment in the liver by additional mechanisms such as modulation of intracellular signaling cascades and metabolic pathways. Several lines of evidence suggest that HCV replication, assembly and entry are closely intertwined with and modulate the hepatic glucose, lipid and cholesterol metabolism [13,14]. Clinically, these modulations manifest themselves in the form of insulin resistance, fatty liver and hypobetalipoproteinemia [15]. Importantly, these pathologies are driven by, and in turn, augment oxidative stress and thus favor inflammation, fibrosis development and disease progression towards cirrhosis and HCC. *Clement et al.* shed light on the interplay between hepatic glucose and lipid metabolism, oxidative stress and inflammation, their potential roles in the HCV life cycle and in the associated pathologies, while a review by Bartosch *et al.* discusses the importance of lipids for HCV infection and the cell entry process in particular.

### Hepatocarcinogenesis

Viral eradication is the most efficient strategy to reduce the risk of HCC development in HBV and HCV patients with chronic liver disease. However, this does not apply to patients who have already developed cirrhosis. Once cirrhosis has occurred, despite the eradication of the cause of liver disease, the risk of HCC is increased since cirrhosis is considered as a preneoplastic state. At this stage of the disease, efficient antiviral treatment decreases, but does not ablate the risk of HCC development [16]. The molecular mechanisms underlying hepatocarcinogenesis remain far from understood, including the responsible etiological factors, the marked genetic and epigenetic heterogeneity, as well as the cell types at the origin of transformation. A review by Benhenda *et al.* discusses the molecular mechanisms underlying hepatitis virus-induced hepatocarcinogenesis, while Merle *et al.*, focus on oncogenic pathways that are activated particularly at late stages of the transformation process in virus and non-virus induced hepatocarcinogenesis.

### Limitations to the investigation of HBV and HCV

A better understanding of the interactions between HBV and HCV and the hepatocytes as well as other liver resident cell types and the immune system is warranted in order to understand the pathologies associated with chronic hepatitis virus infection and how they may predispose to cancer. Two major obstacles hinder the investigation of HBV- and HCV-host cell interactions. The first is the fact that the liver is a highly specialized and structured organ, with physiological functions that depend on a defined liver architecture and proper cell differentiation and polarization. The recapitulation of these features *in vitro* is difficult, and thus *in vitro* replication systems for HBV and HCV have so far relied heavily on tissue culture adapted, transformed cell lines. Secondly, HBV and HCV display a liver tropism that is restricted to very few species apart from humans, which has made the development of animal model systems difficult. Meuleman *et al.* summarize the animal models that have been developed over the years and that have now become available to study HCV infection *in vivo*, and a review by Benhenda *et al.* mentions animal systems available for the study of HBV.

## Summary

Hepatocarcinogenesis is, and will continue to be a major worldwide health problem. With chronic HBV and HCV infections being responsible for a significant proportion of HCC cases, the development of new and relevant cell culture and animal models to study the interactions of HBV and HCV with their host and the development of efficient means to combat chronic infections will remain major tasks to tackle. This special issue gives an overview of our current state of knowledge in respect to the basic biology of these viruses, as well as the clinical and therapeutic options that have been, and are being developed, and highlights the major current technical and biological limitations that the field needs to overcome.
